# Retrospective Analysis of Arthroscopic Superior Labrum Anterior to Posterior Repair: Prognostic Factors Associated with Failure

**DOI:** 10.1155/2013/125960

**Published:** 2013-03-25

**Authors:** Rachel M. Frank, Shane J. Nho, Kevin C. McGill, Robert C. Grumet, Brian J. Cole, Nikhil N. Verma, Anthony A. Romeo

**Affiliations:** ^1^Division of Sports Medicine, Department of Orthopaedic Surgery, Rush University Medical Center, 1611 West Harrison Street, Suite 300, Chicago, IL 60612, USA; ^2^Department of Diagnostic Radiology, Henry Ford Hospital, Detroit, MI, USA

## Abstract

*Background*. The purpose of this study was to report on any prognostic factors that had a significant effect on clinical outcomes following arthroscopic Type II SLAP repairs. *Methods*. Consecutive patients who underwent arthroscopic Type II SLAP repair were retrospectively identified and invited to return for follow-up examination and questionnaire. Statistical analysis was performed to determine associations between potential prognostic factors and failure of SLAP repair as defined by ASES of less than 50 and/or revision surgery. *Results*. Sixty-two patients with an average age of 36 ± 13 years met the study criteria with a mean followup of 3.3 years. There were statistically significant improvements in mean ASES score, forward elevation, and external rotation among patients. Significant associations were identified between ASES score less than 50 and age greater than 40 years; alcohol/tobacco use; coexisting diabetes; pain in the bicipital groove on examination; positive O'Brien's, Speed's, and/or Yergason's tests; and high levels of lifting required at work. There was a significant improvement in ASES at final followup. *Conclusions*. Patients younger than 20 and overhead throwers had significant associations with cases requiring revision surgery. The results from this study may be used to assist in patient selection for SLAP surgery.

## 1. Introduction

Superior labrum anterior to posterior (SLAP) lesions may occur in the athletic and working populations and represent a common source of shoulder pain in these patients. With a prevalence of approximately 6% [1, 2] in the general population and even higher in the active, military population [3], the classification, mechanisms of injury, and surgical treatment of these somewhat common injuries have been thoroughly described in the literature. The arthroscopic surgical management of SLAP tears has evolved over the years and varies depending on the type of tear, ranging from simple excision to debridement to formal repair with and without concurrent treatment to the long head of the biceps tendon (tenotomy or tenodesis). 

Advancements in imaging, techniques, and instrumentation have improved our ability to perform all-arthroscopic SLAP repairs; yet significant controversy regarding diagnosis, operative indications, and treatment technique continues to exist. To further complicate the matter, substantial anatomic variation has been demonstrated in this region of the shoulder [4, 5] which may sometimes cause a nonpathologic labrum to appear injured, leading to inappropriate or even unnecessary surgery. Clinical outcomes after SLAP repair have been reported as good to excellent in 63%–100% of patients; thus, up to approximately one-third of patients are still dissatisfied after SLAP repair [6–13]. While recently some authors have analyzed the correlation between presenting symptoms and mechanism of injury [14, 15], it is unknown if there are any variables or factors that can predict the potential success or failure of SLAP repair for a given patient. The purpose of the present study was to report on potential prognostic factors that may have a significant effect on clinical outcomes following arthroscopic repair of Type II SLAP tears.

## 2. Materials and Methods

Between 2004 and 2006, all patients with a Type II SLAP lesion repair performed by senior fellowship-trained surgeons at a single institution were retrospectively identified. Inclusion criteria for participation in the study were followup greater than two years, and exclusion criteria were patients undergoing treatment for a SLAP tear other than Type II (labral fraying with detached biceps tendon anchor). Patients were not excluded for having concomitant procedures including rotator cuff repair, biceps tenodesis, subacromial decompression, or acromioplasty. Our institutional review board approved the study proposal, and informed consent was obtained for all patients prior to data collection. 

Patients that met the study criteria were contacted by telephone and invited to return for followup examination and questionnaire (Table 1). At baseline and final followup, shoulder functional outcome was measured using validated, shoulder-specific outcome scores including the Simple Shoulder Test (SST), American Shoulder and Elbow Score (ASES), Single Assessment Numeric Evaluation (SANE) score, and Visual Analogue Score (VAS). Each questionnaire also contained the Short Form-12 (SF-12) health status survey (Table 1). Physical examination by a single orthopaedic research fellow was performed independent from the operating surgeon, with all components of the examination performed on both shoulders. The examination consisted of active and passive ranges of motion measured with a goniometer, including active forward elevation in the scapular plane, external rotation at the side, and internal rotation behind the back. A dynamometer (Commander Muscle, JTech Medical, Salt Lake City, UT, USA) was used to test strength in both forward elevation and external rotation at the side. In addition, several diagnostic clinical tests, including the O'Brien's [16] test, Kibler test [17], Speed's test [18], Yergason's test [19], Compression-rotation test [2], Apprehension test, and Relocation test [20], were performed.

Intraoperative information, including diagnostic information, number and type of anchors used, concomitant procedures performed at the time of surgery, labral pathology (location and size), chondral lesions (location, size, and depth), and biceps pathology (none, incomplete or complete tear) was also collected.

### 2.1. Surgical Technique

**Figure 1:**
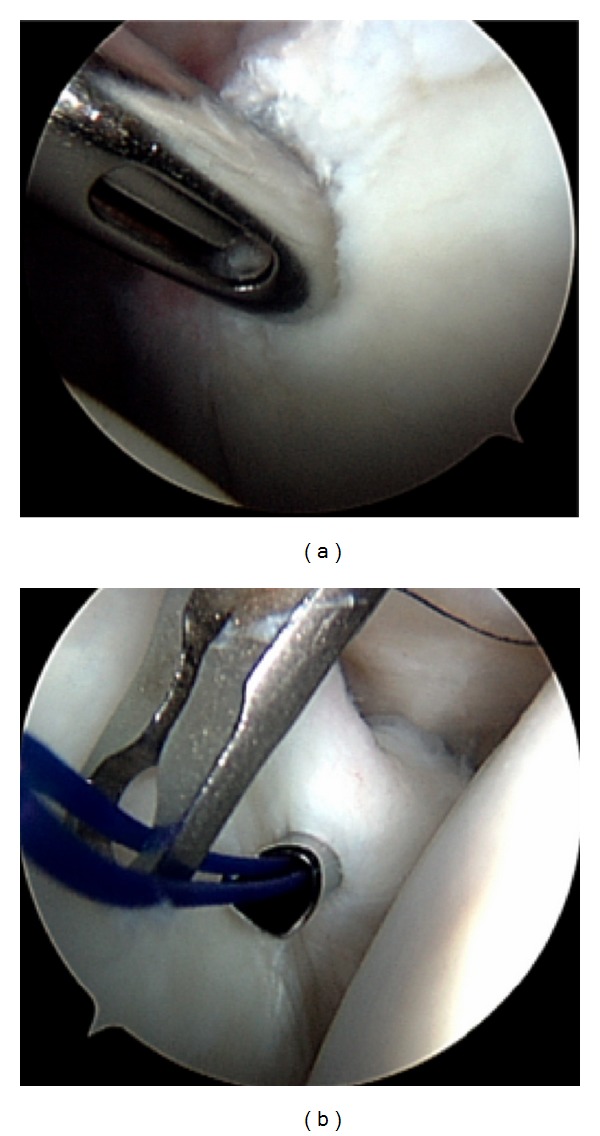
Arthroscopic figures demonstrating surgical technique of SLAP repair: (a) a hooded arthroscopic burr is used to debride the superior glenoid to bleeding cancellous bone to facilitate labral healing; (b) passage of no. 1 PDS suture posterior to the biceps tendon and underneath the labrum.

Patients in our institution undergo a combination of general anesthesia and an interscalene block for postoperative pain control. The patient is placed in either the beach chair or lateral decubitus position with shoulder suspension. The posterior portal is created 3 cm inferior and in line with the acromial angle, and a 30-degree arthroscope is introduced into the glenohumeral joint. The anterior portal is established high in the rotator interval with an outside-in technique utilizing a spinal needle. A 8.25 mm cannula is placed for instrumentation. A diagnostic arthroscopy is performed to determine the extent of the SLAP lesion as well as to assess for other concomitant pathology. Once a Type II SLAP tear is confirmed, a hooded arthroscopic burr is used to debride the superior glenoid to bleeding cancellous bone to facilitate labral healing. A spinal needle is inserted adjacent to the lateral acromial edge aiming toward the superior glenoid just below the biceps origin. A small skin incision is made, and the spear and trocar for the suture anchor are introduced into the glenohumeral joint at a 45-degree angle just medial to the glenoid articular surface. A 3.0 mm suture anchor (BioComposite SutureTak, Arthrex, Inc., Naples, FL) is positioned at 12 o'clock. Through the anterior portal, a 45 degree curved suture shuttle device (Spectrum, Linvatec, Key Largo, FL) loaded with a no. 1 PDS suture is passed posterior to the biceps tendon and underneath the labrum. The PDS suture is advanced into the joint, and the suture shuttle device is carefully withdrawn back out of the anterior cannula. Using the same cannula, a suture retriever is used to grasp both the passed PDS and the FiberWire from the suture anchor and withdraws both of them to prevent suture tangling. The PDS suture is the tied to the FiberWire suture outside the joint, and the PDS limb still attached to the suture shuttle device is pulled to shuttle the FiberWire underneath the labrum and back out of the anterior cannula. The other FiberWire limb is pulled out of the anterior cannula with a crochet hook. The FiberWire suture that is passed through the labrum is the postlimb, and an arthroscopic knot using five reverse half hitches with alternating posts is tied with the knot on top of the superior labrum and away from the articular surface. Using the same steps as previously described, another suture anchor can be placed posterior to the initial anchor at 10 o'clock (right shoulder) depending on the posterior extent of the SLAP tear (Figure 1).

### 2.2. Standard Postoperative Rehabilitation Protocol for All Patients

All patients followed the same standardized rehabilitation protocol postoperatively. For the first 6 weeks, the shoulder was immobilized, with passive- and active-assisted ranges of motion permitted, including motion up to 40° of external rotation and 140° of forward flexion. From 6 to 12 weeks, the patient was advanced to active range of motion. The final 12 weeks focused on rotator cuff strengthening and conditioning. All patients were released to full activity after 6 months. 

### 2.3. Indicators of Surgery Failure

Indicators of surgery failure included revision surgery on the ipsilateral shoulder related to the capsule and/or labrum, ASES less than 50, complications (stiffness, recurrent instability), and/or poor patient satisfaction.

### 2.4. Statistical Analysis

Statistical analysis was performed utilizing both parametric and nonparametric testing methods using SPSS software (SPSS, Chicago, IL, USA). Descriptive analysis consisted of frequencies and percentages for discrete data and means and standard deviations for continuous data. Paired student's *t*-test were used comparing preoperative measures with corresponding postoperative measures at final followup. Contingency table analysis using Fisher's Exact test was used for identifying correlations between potential risk factors and outcome measures. Significance was set at *P* < 0.05 for all tests.

## 3. Results

Sixty-two patients with an average age of 36 ± 13 years were available for followup from an original cohort of 100 consecutive patients (62% followup rate). The average followup duration was 3.3 years (range: 2.0 to 5.0). Forty-six patients (74%) were male, and 16 (26%) were female. Eleven patients reported either active or prior tobacco history. In addition to SLAP repair, several patients also underwent concomitant Bankart repair (*n* = 9), rotator cuff tear repair (*n* = 10), acromioplasty (*n* = 8), distal clavicle resection (*n* = 2), and biceps tenodesis (*n* = 9).

There were statistically significant improvements in the average ASES (preop: 64.8 ± 19; postop: 83.9 ± 18.3; *P* < 0.001), SST (preop: 8.6 ± 2.9; postop: 10.3 ± 2.3, *P* = 0.004) and VAS (preop: 3.3 ± 2.3; postop: 1.6 ± 1.9, *P* < 0.001) scores. The mean postoperative SANE score, indicating the patient's overall assessment of their shoulder function, was 86.9 ± 16.4. 

There was a statistically significant improvement in average forward flexion (preop: 156 ± 34°; postop: 172 ± 14°, *P* = 0.005) when comparing preoperative values to those postoperatively. Similarly, there were clinically relevant improvements in average, external rotation (preop: 66 ± 19°; postop: 70 ± 12°, *P* > 0.05) and abduction (preop: 155 ± 34°; postop: 169 ± 66°, *P* > 0.05); however, these results were not statistically significant.

Patients with a postoperative ASES of less than 50 and/or those who went onto revision surgery were considered failures. There were a total of 5 patients (8.1%) with a postoperative ASES less than 50. In addition, a total of 5 other patients (8.1%) went onto receive revision shoulder surgery. Thus, the overall failure rate was 16.2% for the entire cohort. 

There was a significant association identified between patients with an ASES less than 50 and several factors, including age greater than 40 years (*P* = 0.005), alcohol use (*P* = 0.033), tobacco use (*P* = 0.002), and diabetes (*P* < 0.001). Associations between physical examination maneuvers, including pain in the bicipital groove on examination (*P* < 0.001), positive O'Brien's test (*P* = 0.002), positive Speed's test (*P* < 0.001), and positive Yergason's test (*P* = 0.015) were also seen with ASESs less than 50. Finally, there was a significant association between ASES less than 50 and high levels of lifting required at work (*P* = 0.004). 

The 5 revision surgeries included capsular release (softball injury), 270-degree labral repair after traumatic retear of labrum (baseball injury), two revision SLAP repairs (both baseball injuries), and debridement to SLAP repair (wrestling injury). Two out of 3 patients under 20 years old were revised, as compared to 3 out of 35 patients aged 20 years and older. Four out of 10 patients who were throwers were revised, as compared to 0 out of 29 nonthrowing patients. A significant association was identified between patients requiring revision surgery and age less than 20 years (*P* = 0.035) as well as preoperative participation in throwing activities (*P* < 0.001). Overhead throwers were defined as patients who use their arms in an overhead position, including but not limited to baseball players (including pitchers), football players, swimmers, and tennis players. 

## 4. Discussion

Although the technical aspects of arthroscopic repair of Type II SLAP tears have been well described, the clinical decision making may not be as apparent. There may be a certain subset of patients that have suboptimal clinical outcomes after surgical fixation unstable SLAP lesions. The present study suggests that arthroscopic repair of Type II SLAP tears results in a significant improvement in shoulder functional outcome and range of motion; however, there are a number of prognostic factors that may have a higher association with clinical failure. The principle findings of this study include the following: (1) when using revision surgery as an indicator of failure, the prognostic factors most associated with failure were overhead throwers and age less than 20 years, and (2) when using ASES less than 50 as an indicator of failure, the prognostic factors most associated with failure were age greater than 40 years, heavy laborers, users of tobacco and/or alcohol, diabetics, and/or patients who present with persistent anterior shoulder pain (symptoms consistent with persistent SLAP lesion or bicipital groove tenderness).

Using a poor ASES as a reflection of overall poor shoulder function, the results from the present study suggest that patients more likely to fail SLAP repair are older than 40 years old, heavy laborers, users of tobacco and/or alcohol, diabetics, and/or patients who present persistent SLAP or bicipital groove pain (tenderness over the long head of the biceps tendon, positive O'Brien's test, positive Speed's test, and/or positive Yergason's sign). These are the type of patients that one might expect to have a poor outcome due to persistent bicipital symptoms and not necessarily due to the SLAP tear or repair itself. In patients over 40 years old with a Type II SLAP lesion, the decision of whether to perform a SLAP repair, biceps tenodesis, or SLAP repair with a biceps tenodesis remains unclear as the true etiology of symptoms in this specific patient population is extremely difficult to determine clinically. Although the cohort in the present study only evaluated patients who have had SLAP repairs, these patients may have had improved shoulder functional outcome with a biceps tenodesis with or without a SLAP repair [4, 21]. Boileau et al. [21] recently studied the clinical outcomes following arthroscopic biceps tenodesis using interference screws as an alternative to repair of isolated unstable Type II SLAP defects. The authors found that patients were subjectively more satisfied and had a significantly higher rate of return to previous level of activity in the biceps tenodesis group as compared to the SLAP repair group, including patients participating in overhead sport. Interestingly, in this study, the patients in the biceps tenodesis group were significantly older with a mean age of 52 years (range: 28–64) compared to the SLAP repair group with a mean age of 37 years (range: 19–57) (*P* < 0.001), which clearly may be a contributing factor to the success of the biceps tenodesis procedure in this cohort. 

When using revision surgery as the definition of a failed SLAP repair, age under 20 years was a significant (negative) prognostic factor. Based on these results, it is evident that greater proportions of patients under 20 years old had to be revised compared to their comparison groups (patients over 20 years old). It is possible that young patients who had SLAP repairs are less likely to tolerate these repairs, potentially due to postoperative stiffness and/or reinjury. 

As proposed by Burkhart and Morgan [26] in 2001, it is possible that the mechanism of SLAP injury in overhead athletes, notable baseball pitchers, is actually related to the acceleration phase of throwing when the shoulder is in a position of extreme abduction and external rotation. Overhead athletes with high pitching/throwing volumes may develop posteroinferior shoulder stiffness, causing a deficit in internal rotation range of motion and subsequent stiffness, also known as the “dead arm syndrome” as coined by Rowe [27]. This becomes problematic when the athlete acquires a SLAP lesion, as unlike in a healthy shoulder, the patient is unable to compensate for their internal rotation deficit with a gain of external rotation. In an outcomes study of SLAP repairs comparing overhead athletes to nonoverhead athletes, Kim et al. [11] found that nonoverhead athletes had significantly better outcomes when using UCLA scores and return to preinjury level of activity as outcomes assessments. Specifically, the authors reported that only 22% of overhead athletes returned fully to their preinjury level of activity, as compared with 63% of the nonoverhead athletes. Interestingly, Pagnani et al. [28] found that 12/13 athletes (92%) were able to return to their preinjury level of overhead activity following SLAP repair. Ide et al. [25] found that 36 of 40 (90%) overhead athletes were satisfied with their SLAP repair, with 75% of the athletes returning to their preinjury level of competitiveness. Finally, Yung et al. [22] recently found that overhead athletes required a longer duration of therapy/rehabilitation in order to return to their preoperative level of activity following SLAP repair. Thus, the results represented in the literature are inconsistent, and the reason explaining why overhead athletes may be more likely to be less satisfied or take longer to return to activity after SLAP repair remains largely unknown. 

Recently, Katz et al. [14] performed an analysis of patients with poor outcomes following SLAP repair, with a focus on outcomes following subsequent treatment after the initial poor outcome. Overall, the authors reported that while 68% of their patient cohort was satisfied after initial SLAP failure followed by either surgical or nonoperative therapy, 32% continued to have a suboptimal response. While the authors commented on the number of patients who used tobacco (4) and had a history of diabetes (2), no statistical analysis was performed in attempt to correlate these and other similar demographic and social factors with a potential prognostic significance. In addition, there are a number of additional studies available that report on outcomes following SLAP repair, several of which report associations between poor outcomes and specific factors (Table 2 [6–9, 11–15, 21–25, 29]). 

This study had several limitations, most notably its retrospective nature, lack of control group, and followup rate of 62%. Multiple attempts were made to contact all of the 100 consecutive patients in the initial cohort, and unfortunately due to missing and/or incorrect contact information, several patients were unable to be reached. Another limitation is the number of concomitant procedures performed in our patient population. One major difficulty with treating shoulders with multiple injuries is understanding which lesions are truly symptomatic and which are simply incidental. As discussed by Boileau et al. [21] and Kim et al [30], it is impossible to know if patients who had a successful outcome following, for example, both SLAP and rotator cuff repair, benefited more from one of the repairs versus the other, or if both were truly needed to produce a successful outcome. Subgroups of patients undergoing concomitant procedures could not be statistically analyzed secondary to the small number of patients undergoing these procedures as well as the overlap between patients undergoing more than one concomitant procedure. Another limitation is the lack of followup imaging, which would have provided another objective outcome as to whether or not the SLAP repairs remained intact. 

This study also had several strengths. To our knowledge, this is the first study that discusses the prognostic factors affecting the clinical outcome after SLAP repair. All patients of this relatively large cohort completed questionnaires utilizing validated, shoulder-specific outcomes surveys. Additionally, all patients were examined by a single, blinded orthopaedic research fellow. There were four fellowship-trained orthopaedic surgeons, in either sports medicine or upper extremity surgery, performing all procedures at a single institution, allowing the results to be generalizable to other surgeons who focus on the shoulder. Finally, the rehabilitation protocol utilized was standardized for all patients. 

## 5. Conclusion

Overall, patient selection in SLAP repairs can be difficult, and the results from this study may be used to assist with patient selection for SLAP surgery and can help predict which patients might benefit from SLAP repair and which are less likely to experience significant improvement. Further long-term studies necessary to determine the natural history of SLAP repair as well as to determine factors that may be associated with improved surgical outcomes.

## Figures and Tables

**Table 1:** Prognostic factors asked on post-operative questionnaire.

Factor	Possible Responses
Age	Years
Tobacco history	Yes or no
Preoperative pain	Yes or no
Anti-inflammatory use	Yes or no
Narcotic use	Yes or no
Extremity	Right or left
Dominant extremity	Yes or no
Trauma	Yes or no
Mechanism or injury	Sports, motor vehicle accident, fall, traction, insidious
Sport	
Level of sports participation	Professional, collegiate, high school, recreational, none
Thrower	Yes or no
Overhead athlete	Yes or no
Collision sport	Yes or no
Level of work	Very heavy, heavy, medium, light, sedentary
Worker's compensation	Yes or no
History of dislocation	Yes or no
History of subluxation	Yes or no
Pre-operative O'Brien's test	Positive, negative, equivocal
Pre-operative biceps load II test	Positive or negative
Pre-operative Compression-rotation	Positive or negative
Pre-operative Kibler test	Positive or negative
Pre-operative bicipital groove tenderness	Yes or no
Pre-operative Speed's test	Positive or negative
Pre-operative Yergason's test	Positive or negative
Pre-operative apprehension test	Positive or negative
Pre-operative relocation test	Positive or negative

**Table 2:** Outcomes and potential contributing factors following arthroscopic SLAP lesion repair.

Authors (reference)	Number of patients	Clinical outcome measures	Outcomes	Potential factors
Katz et al., 2009 [14]	40 shoulders (39 patients)	SST, patient satisfaction	71% of those with poor outcome dissatisfied with conservative treatment	Not discussed
Brockmeier et al., 2009 [6]	47	ASES, L'Insalata	87% good to excellent	Higher outcomes after traumatic etiology
Boileau et al., 2009 [21]	10 (15 others with BT)	Constant, patient satisfaction	(i) Constant score 65 → 83 (ii) 60% dissatisfied (iii) 4 overall failures converted to BT	Not discussed
Yung et al., 2008 [22]	16	UCLA, physical exam	31% excellent, 44% good, 25% poor	Overhead athletes required longer time to RTP
Park et al., 2008 [15]	24	UCLA, VAS	(i) UCLA: 22.7 → 29.9 (ii) VAS: 6.4 → 2.1	Mechanism of injury did not impact outcomes
Oh et al., 2008 [23]	25 (58 total in study, only 25 with isolated SLAP lesions)	VAS, ASES, UCLA, SST, constant	Significant improvements: (i) VAS pain: 1.8 (ii) ASES: 84.1 (iii) UCLA 32.6 (iv) SST: 94.7 (v) VAS: 8.9	Not discussed
Voos et al., 2007 [13]	30 (combined RCT with SLAP or Bankart)	ASES, L'Insalata	(i) 90% good to excellent (ii) 77% return to play (iii) 2 recurrent RCT	Not discussed
Funk and Snow, 2007 [24]	18	Satisfaction, time to RTP	89% satisfaction	Isolated SLAP lesions had quickest return to play
Enad et al., 2007 [9]	27 (15 with isolated tears), military population	ASES, UCLA	Excellent in 4, good in 20, fair in 3 96% return to duty	Higher outcomes scores in pts with concomitant diagnosis
Coleman et al., 2007 [8]	50 (16 with concomitant acromioplasty)	ASES, L'Insalata,	(i) 65% good to excellent in SLAP only group (ii) 81% good to excellent in acromioplasty group	Not discussed
Cohen et al., 2006 [7]	39	ASES, L'Insalata,	(i) 71% satisfied (ii) 41% with continued night pain	Athletes and pts with rotator cuff piercing with worse outcomes
Ide et al., 2005 [25]	40, all overhead athletes	Modified Rowe	(i) Rowe: 27.5 → 92.1 (ii) 75% return to preinjury level of activity	Traumatic etiology with better return to activity than overuse etiology
Kim et al., 2002 [11]	34	UCLA	(i) 94% satisfied (ii) 91% return to preinjury level	Overhead sports with lower ASES (*P* = 0.024) and lower return to preinjury level (*P* = 0.015)
O'Brien et al., 2002 [12]	31	ASES, L'Insalata	(i) 52% return to preinjury level (ii) L'Insalata: 87 (iii) ASES: 87.2	Not discussed

Abbreviations: SLAP: superior labrum anterior to posterior; BT: biceps tenodesis; ASES: American Shoulder and Elbow Society; UCLA: University of California Los Angeles; SST: Simple Shoulder Test; VAS: Visual Analog Scale; RCT: rotator cuff tear; RTP: return to play.
